# Perplexities of treatment resistence in eating disorders

**DOI:** 10.1186/1471-244X-13-292

**Published:** 2013-11-07

**Authors:** Katherine A Halmi

**Affiliations:** 1 New York Presbyterian Hospital, Westchester Division, 21 Bloomingdale Rd, Whites Plains, NY 10605, USA

**Keywords:** Treatment resistance, Anorexia nervosa, Bulimia nervosa, Binge eating disorder

## Abstract

**Background:**

Treatment resistance is an omnipresent frustration in eating disorders. Attempts to identify the features of this resistance and subsequently develop novel treatments have had modest effects. This selective review examines treatment resistant features expressed in core eating disorder psychopathology, comorbidities and biological features. Novel treatments addressing resistance are discussed.

**Description:**

The core eating disorder psychopathology of anorexia nervosa becomes a coping mechanism likely via vulnerable neurobiological features and conditioned learning to deal with life events. Thus it is reinforcing and ego syntonic resulting in resistance to treatment. The severity of core features such as preoccupations with body image, weight, eating and exercising predicts greater resistance to treatment. Bulimia nervosa patients are less resistant to treatment with treatment failure related to greater body image concerns, impulsivity, depression, severe diet restriction and poor social adjustment. For those with binge eating disorder overweight in childhood and high emotional eating predicts treatment resistance. There is suggestive data that a diagnosis of an anxiety disorder and severe perfectionism may confer treatment resistance in anorexia nervosa and substance use disorders or personality disorders with impulse control problems may produce resistance to treatment in bulimia nervosa. Traits such as perfectionism, cognitive inflexibility and negative affect with likely genetic influences may also affect treatment resistance. Pharmacotherapy and novel therapies have been developed to address treatment resistance. Atypical antipsychotic drugs have shown some effect in treatment resistant anorexia nervosa and topiramate and high doses of SSRIs are helpful for treatment of resistant binge eating disorder patients. There are insufficient randomized controlled trials to evaluate the novel psychotherapies which are primarily based on the core psychopathological features of the eating disorders.

**Conclusion:**

Treatment resistance in eating disorders is usually predicted by the severity of the core eating disorder psychopathology which develops from an interaction between environmental risk factors with genetic traits and a vulnerable neurobiology. Future investigations of the biological features and neurocircuitry of the core eating disorders psychopathology and behaviors may provide information for more successful treatment interventions.

## Introduction

Treatment resistance is a common feature of eating disorders documented by poor response rates in many treatment trials. Studies of predictors of response to treatment have shown varying results depending on eating disorder diagnosis and definitions of response and recovery [1]. A literature search from years 2000 to 2012 using the terms treatment resistance, anorexia nervosa (AN), bulimia nervosa (BN), binge eating disorder (BED), and eating disorders yielded 38 papers from Pub Med and 26 papers from Psych Info. In the overwhelming majority of these papers the term treatment resistance was used interchangeably with chronicity of illness or difficult to treat. There were also multiple definitions of treatment failure including no definition. The author decided not to present a comprehensive review of “treatment resistance “but rather mention those articles with salient relevant features of the author’s interest in three areas; core eating disorder psychopathology, comorbidity, and biological features. A variety of novel psychotherapies addressing “treatment resistance” in AN have been developed. All of these need further efficacy and effectiveness trials and are referred to briefly as are some pharmacotherapies for AN. A few treatment studies with adequate sample sizes addressing “resistant “patients with BN and BED are presented along with the author’s suggestions.

## Review

### Core eating disorder psychopathology

Treatment resistance is especially prominent in anorexia nervosa patients who often deny their fear of gaining weight and the seriousness of their illness. Many female adolescents with AN have stated openly they do not wish to develop into a mature female body and are fearful of becoming independent of their family [1]. For many AN becomes a coping mechanism to deal with adverse experiences. It provides an escape from aversive developmental (maturity) issues and distressing life events often of an interpersonal nature. Changing their behavior is an overwhelming and terrifying notion to the anorexia nervosa patient. Certain developmental features are common in anorexia nervosa. The majority of these patients have had a lack of experience to foster personal independence [2]. This has produced a sense of personal ineffectiveness and poor self-esteem. Many of these patients also have a social ineffectiveness, which makes them feel ill at ease in dealing with their peers and with life crises. They often have problems of developmental transitions from the prepubertal state through puberty to a mature adult. Their immaturity and autonomy fears are expressed in the form of refusing to separate from their parents. A plausible hypothesis is that their preoccupations with body image, weight, eating and exercising provide a distraction for dealing with distressing life events. In addition, the behavior of their illness gives them a feeling of control and elevates their self-esteem. The mental changes occurring from severe dieting and emaciation further augment treatment resistance. These symptoms of emotional instability, irritability and loss of concentration make it more difficult for the patient to engage in meaningful psychotherapy that results in behavior change. Severity of core eating disorder psychopathology usually predicts greater resistance to treatment in anorexia nervosa patients [3].

Treatment resistance in bulimia nervosa should be able to be inferred from studies of predictors of therapy response. Unfortunately, different studies have found different sets of predictors both for treatment outcome and for attrition. Predictors identified as statistically significant in one study were not found significant in others. This may be due to several factors including the type of therapy, the mode of delivery (i.e., individual or group format, outpatient or inpatient treatment), and the characteristics of the population of bulimic subjects studied. Many studies have had too few subjects to reliably identify outcome predictors and the definition of treatment success has varied from abstinence from binge eating and purging to no longer meeting criterion of DSM-IV diagnostic criteria. Pretreatment variables and the methods of assessing treatment outcome have varied among the studies. The fact that bulimia nervosa patients are less resistant to treatment than those with anorexia nervosa is demonstrated by the fact there many more randomized controlled trials for treatment of bulimia nervosa compared with anorexia nervosa, a condition in which few people are willing to enter the trials and when they do the dropout rate and lack of commitment to treatment is considerably greater. One study of 194 women with bulimia nervosa showed those with the treatment resistance (treatment failure) had greater concerns about shape and had greater impulsivity than those who responded to treatment. Non-responders to the immediate end of treatment were also more likely to have current depression, a lower body mass index indicating severe dietary restriction and poor social adjustment [4].

The problem of different studies showing contradictory results for predictors of treatment resistance in BN is present as well as in BED. One study with a large sample of 144 individuals with binge eating disorder found a history of overweight during childhood and high emotional eating were predictors of treatment resistance [5]. In two recent studies severity of body image disturbance and shape concerns were related to treatment compliance and resistance [6,7].

### Psychiatric and psychological comorbidity

Studies presented in this section were chosen for large sample size or the mention of specific traits or concepts. The most comprehensive psychiatric comorbidity study is from the U.S. National Survey Replication [8]. In this study at least one lifetime comorbid psychiatric DSM-IV disorder was present in 56.2% of anorexia nervosa participants, 94.5% of those with bulimia nervosa, 78.9% of those with binge eating disorder, 63.6% with sub threshold binge eating disorder, and 76.5% with any binge eating. Affective disorders which are the most prevalent of the comorbid psychiatric disorders associated with eating disorder were not shown to influence the long term outcome response to treatment in bulimia nervosa [9] or to effect treatment acceptance or completion in anorexia nervosa [3]. The rates of anxiety disorders were similar in 97 individuals with anorexia nervosa, 282 with bulimia nervosa and 293 with anorexia nervosa binge-purge subtype in a large genetic study [10]. Two-thirds of the participants in this study reported one or more anxiety disorders in their lifetime with the most common diagnosis being obsessive-compulsive disorder, 41% and social phobia, 20%. In the majority of these patients the onset of anxiety disorders occurred in childhood before the emergence of their eating disorder. In this study those individuals who had a lifetime diagnosis of an anxiety disorder and were currently ill with an eating disorder had more symptoms of anxiety, harm avoidance, obsessionality and perfectionism suggesting that a diagnosis of anxiety disorder and more severe symptoms of the traits mentioned may confer treatment resistance. Substance use disorders (alcohol and or drug) occur in 40 to 50 percent of disorders with binge eating. In these eating disorder subtypes substance use disorders are associated with a high prevalence of major depression, anxiety disorders, and cluster B personality disorders [11]. The high degree of multiple comorbidities with substance use disorders and eating disorders makes it difficult to determine the effect that a substance use disorder may have on treatment resistance. Personality disorders are also highly prevalent in the eating disorder population. In one large comprehensive study [12] 69% of the patients had a least one personality disorder and 31% of the bulimic subgroups had cluster B impulsive disorders of which the most prominent was borderline personality disorder present in 25%. The prevalence of cluster C anxious personality disorders was present in 30% of eating disorder patients and did not vary according to eating disorder subtype. A review of bulimia nervosa studies [13] concluded that personality disorders marked by problems with impulse control were associated with a worse prognosis in these patients and thus suggesting an association with treatment resistance.

There is some indication that personality traits may influence response to treatment and treatment resistance. Perfectionism is a personality trait initially identified with anorexia nervosa. In one study of 322 women with a history of anorexia nervosa greater perfectionism was associated with lower body weight, greater prominence of eating preoccupations and rituals and a diminished motivation to change, the latter implying a greater resistance to treatment [14]. There is an indication that temperament features may also affect responsiveness to treatment in eating disorders. Diagnostic crossover from anorexia nervosa to bulimia nervosa and visa versa in one large sample study was consistently associated with low self-directiveness [15]. There is also a suggestion that the severity of negative affect may influence treatment responsiveness in binge eating disorder [16]. In a study of 74 individuals with eating disorders including anorexia nervosa, bulimia nervosa and eating disorders not otherwise specified, those patients with a combination of low self compassion and high fear of self compassion at baseline had significantly poorer treatment responses [17]. In a response to a stressful speech task, recovering anorectic patients demonstrated greater negative emotional responses compared with controls. The author suggested that the persistence of a negative affect with distress following recovery may place these patients at risk for relapse and this may also influence anorectic patient’s resistance to treatment [18]. Difficulties in cognitive flexibility are characteristics of patients with anorexia nervosa and may account for treatment resistance in these patients [19]. Cognitive remediation therapy has been proposed as an adjunctive treatment for patients with anorexia nervosa. On a scale measuring existential well being anorexia nervosa participants were found to score significantly lower than age matched controls [20]. The author’s suggested that anorexia is a coping strategy that provides a sense of meaning and identity. This however may cause more existential anxiety since these individuals would have limited mechanisms for dealing with this anxiety and make them resistant to treatment efforts.

In a treatment study of binge eating disorder those patients who responded to treatment with a behavioral change had the following 6 attributes; 1) they strongly wanted and intended to change for clear, personal reasons, 2) they faced a minimum of obstacles to change, 3) the patient had the requisite skills and self-confidence to make the change, 4) the patient felt positive about the change and believed it would result in meaningful benefits, 5) the patient perceived the change as congruent with self image and social group norms, 6) the patient received encouragement and support to change from valued persons [21].

### Biological features

Considerable evidence for altered brain serotonin and dopamine function from brain imaging is present for AN and BN. Although these alterations have not been shown to be directly related to treatment resistance, there is evidence they are related to psychological and behavioral features whose severity is associated with treatment resistance. For example, serotonin transporter function has been related to extremes of impulse control in BN [22]. A positron emission tomography (PET) study found interactions between D2/D3 receptor and serotonin transporter binding were related to harm avoidance. The latter is a measure of inhibition and anxiety [23]. Another study showed increased serotonin 1A receptor binding in recovered bulimics and this positively correlated to harm avoidance in specific brain areas [24]. A later study showed ill and recovered BN have altered serotonin transporter binding which the authors suggest may influence responses to medication [25].

Psychometric studies have linked AN and BN to a cluster of moderately heritable personality and temperamental traits, such as obsessionality, perfectionism, and harm avoidance [26] A linkage analysis of an AN cohort containing extreme high ratings for drive-for-thinness and obsessionality produced a significant linkage at 1q31.1 for those covariates. The allele frequency differences in the GABA receptor SNP, GABRG_1_ was found to be related to levels of trait anxiety in anorexia and bulimia nervosa probands. High anxiety levels are characteristic and present to a greater degree in persistently ill eating disorder probands compared with those who have recovered. Thus, this GABA receptor aberration may be related to treatment resistance [27]. In a study of behavioral profiles of anorexia nervosa probands and their parents, a class was identified of probands with mother – daughter symptom severity for eating disorder psychopathology and anxious/perfectionistic traits. It could be hypothesized that these probands may have a genetic propensity for treatment resistance [28].

### Efforts at treating refractory patients

The literature on treating refractory patients consists mainly of suggestions based on observations of patients. These studies are mostly uncontrolled and with small samples. Over the past 20 years eating disorder hospital treatment has changed from long term treatment to stabilization of acute episodes. For treatment resistant patients this change has been deleterious and not cost effective as shown in one study with readmissions changing from 0% to 27% of total admissions [29]. For anorexia nervosa patients contingencies attached to behavioral goals could be changed or the intensity of treatment increased such as residential treatment with a daily structure in careful monitoring to prevent readmissions. At times involuntary hospitalization with enteral feeding may be necessary. Pharmacotherapies that have shown some effectiveness in treating resistant anorexia nervosa include haloperidol [30] quetiapine and olanzapine [31,32] and Duloxetine [33]. Several novel psychotherapies are being developed for treating resistant anorexia nervosa include the following; 1) Cognitive Behavioral Therapy Extended [34]: this focuses on addressing predisposing and maintaining factors of the eating disorder as well as involving caregivers to support the patient with matters regarding food, eating and psychological factors. Cognitive Remediation Therapy was developed to treat an inflexible thinking style [35]. Modest results were obtained. Another 10 session treatment package that primarily addresses emotion processing difficulties in the self and others and includes strategies to manage emotions and the practice of emotion expression has also had modest results. This therapy is called Cognitive Remediation and Emotional Skills Training (CREST) [36]. Maudsley Model for Treatment of Adults with Anorexia Nervosa (MANTRA) is another form of therapy which addresses rigid thinking styles with perfectionism and obsessive compulsive personality traits and the avoidance of strong emotional responses to others. It includes motivational interviewing and a CBT framework [37]. Community outreach partnership program (COPP) has a goal of improving quality of life and minimizing harm [38]. Specialist supportive clinical management (SSCM) emphasizes support for changes that will improve quality of life as well as physical well being. Its aim is to provide a therapeutic match to the chronic patient’s level of ambivalence [39,40]. It does this by allowing flexibility in the approach. Strober [41], advocates a different paradigm in which management replaces traditional objectives of therapy to support the patient in a palliative holding management of carefully measured intensity. Small steps are taken to partially compensate or cushion the effects of the illness. This is done by assuring the patient weight gain will not be a principle objection of the management approach, encouraging the patient to maintain some type of social activity and involvement in hobbies, intellectual pursuits or activities that allow for feelings of pleasure. It also requires regular physical exams and exploring possibilities of improvement in nutrition. In therapy with treatment resistant anorectics Vanderlinden [42], emphasizes the quality of the therapeutic alliance and the timing of therapeutic strategies as well as focusing less on the content of cognitions and more of the emotional involvement with cognitions. He also emphasizes focusing on dysfunctional cognitions and messages within the family communications and interactions.

In treating resistant bulimia nervosa patients sequential treatment is often effective. In one study [43], 20% of non responders to cognitive behavioral therapy responded to fluoxetine or interpersonal therapy at the same rate. Resistant bulimics may require a partial hospitalization or inpatient program for a short period of stabilization. Additional or separate treatment for comorbid diagnoses with bulimia may be indirectly helpful. Examples are alcoholics anonymous for those with substance abuse or dialectal behavior therapy for those with borderline personality disorders. Cue exposure was effectively used to treat resistant adolescents with bulimia nervosa [44]. The authors emphasize that cue exposure prevented the binge itself whereas exposure response therapy prevents post bingeing behaviors and the latter has not been effective in treating resistant bulimics.

For binge eating disorder resistant patients a recommendation is suggested of high doses of SSRIs or the drug topiramate starting at 25 mg daily and increased by 25 mg weekly [45].

## Conclusions

Trait – related multigenic and neurobiological vulnerability when modulated by environmental risk factors influence the development of eating disorders and may also contribute to treatment resistance. These conditions once established are sustained by conditioned learning and state related pathophysiological changes [46]. Since none of the efforts at treating resistant eating disorder patients have been dramatically effective, future investigations of neurobiological factors and neurocircuitry in eating disorder patients may provide information for more successful treatment interventions.

## Competing interests

The author has no competing interest.

## Pre-publication history

The pre-publication history for this paper can be accessed here:

http://www.biomedcentral.com/1471-244X/13/292/prepub

## References

[B1] Halmi KA Perplexities and provocations of eating disorders J Child Psychol Psychiat 2009 50 163 169 10.1111/j.1469-7610.2008.01983.x 19220599

[B2] Wagner S Halmi KA The sense of personal ineffectiveness in patients with anorexia nervosa: one construct or several? Int J Eat Dis 1987 6 495 505 10.1002/1098-108X(198707)6:4<495::AID-EAT2260060406>3.0.CO;2-I

[B3] Halmi KA Agras WS Crow S Mitchell J Wilson GT Bryson SW Kraemer C Predictors of treatment acceptance and completion in anorexia nervosa Arch Gen Psychiat 2005 62 776 781 10.1001/archpsyc.62.7.776 15997019

[B4] Agras S Crow S Halmi KA Mitchell J Wilson T Kraemer H Outcome predictors for the cognitive – behavioral treatment of bulimia nervosa: data from a multisite study Am J Psychait 2000 157 1302 1308 10.1176/appi.ajp.157.8.1302 10910795

[B5] Ricca V Castellini G Mannucci E Sauro CL Ravaldi C Rotella CM Faravelli C Comparison of individual and group cognitive behavioral therapy for binge eating disorder: a randomized, 3 year follow up study Appetite 2010 55 656 665 10.1016/j.appet.2010.09.019 20870000

[B6] Towell DB Woodford S Reid S Rooney B Towell A Compliance and outcome in treatment – resistant anorexia and bulimia: a retrospective study Brit J Clin Psychol 2001 40 189 195 10.1348/014466501163634 11446240

[B7] Ricca V Castellini G Sauro CL Mannucci E Ravaldi C Rotella F Faravelli C Cognitive – behavioral therapy for threshold and sub threshold anorexia nervosa: a 3 year follow up study Psychother Psycholsom 2010 79 238 248 10.1159/000315129 20502064

[B8] Hudson J Pope H Hiripi E Kessler R The prevalence and correlates of eating disorders in the national comorbidity survey replication Biol Psychiat 2008 2 1 32 10.1016/j.biopsych.2006.03.040 PMC1892232 16815322

[B9] Walsh T Hadigan C Devlin M Gladis M Roose S Long term outcome of antidepressant treatment for bulimia nervosa Am J Psychait 1991 148 12106 12112 10.1176/ajp.148.9.1206 1882999

[B10] Kaye W Bulik C Thornton L Barbarich N Masters K Comorbidity of anxiety disorders with anorexia and bulimia nervosa Am J Psychiat 2004 161 2215 2222 10.1176/appi.ajp.161.12.2215 15569892

[B11] Bulik SM Klump K Thornton L Kaplan A Devlin B Fichter M Halmi KA Strober M Woodside DB Mitchell J Rotondo A Keel P Berrettini W Kaye WH Comorbidity of eating disorders and alcohol use disorders J Clin Pychiat 2004 65 1000 1006 10.4088/JCP.v65n0718 15291691

[B12] Braun DL Sunday SR Halmi KA Psychiatric comorbidity in patients with eating disorders Psychol Med 1994 24 859 867 10.1017/S0033291700028956 7892354

[B13] Keel P Mitchell JE Outcome in bulimia nervosa Am J Psychiat 1997 154 331 335 10.1176/ajp.154.3.313 9054777

[B14] Halmi KA Sunday S Strober M Kaplan A Woodside DB Fichter M Treasurer J Berrettini W Kaye W Perfectionism in anorexia nervosa: variation by clinical subtype, obsessionality and pathological eating disorders Am J Psychiat 2000 157 1799 1805 10.1176/appi.ajp.157.11.1799 11058477

[B15] Tozzi F Thornton L Klump K Fichter M Halmi KA Symptom fluctuations in eating disorders: correlates of diagnostic crossover Am J Psychiat 2005 10 732 740 15800146 10.1176/appi.ajp.162.4.732

[B16] Stice E Agras WS Halmi KA Mitchell JE Wilson GT Subtyping binge eating disordered women along dieting and negative affect dimensions Int J Eat Dis 2001 30 11 27 10.1002/eat.1050 11439405

[B17] Kelly AC Carter JC Zuroff D Borairi S Self-compassion and fear of self-compassion interact to predict response to eating disorders treatment: a preliminary investigation Psychother Res 2012 1 1 13 10.1080/10503307.2012.717310 22917037

[B18] Miller S Erickson S Branon C Steiner H Habitual response to stress in recovering adolescent anorectic patients Child Psychiat Humdev 2009 40 43 54 10.1007/s10578-008-0112-y 18618238

[B19] Tchanturia K Divies H Campbell IC Cognitive remediation therapy for patients with anorexia nervosa: preliminary findings Am Gen Psychiat 2007 6 14 20 10.1186/1744-859X-6-14 PMC1892017 17550611

[B20] Fox AP Leung N Existential well-being in younger and older people with anorexia nervosa – a preliminary investigation Eur Eat Dis Rev 2009 17 24 30 10.1002/erv.895 18759376

[B21] Wadden YA Berkowitz RI Sarier DB Wisnenieski S Steinberg C Benefits of lifestyle modification in the pharmacological treatment of obesity: a randomized trial Arch Intern Med 2001 161 218 227 10.1001/archinte.161.2.218 11176735

[B22] Steiger H Joober R Isreal M Young SN Ng Ying Kin NM Gauvin L The 5HTTLPR polymorphism, psychopathological symptoms and platelet paroxetine binding in bulimic syndromes Int J Eat Disord 2005 37 57 60 10.1002/eat.20073 15690467

[B23] Bailer UF Frank GK Price JC Meltzer CC Becker C Mathis CA Wagner A Barbarich NC Bloss CS Putnam K Schork NJ Gamst A Kaye WH Interaction between serotonin transporter and dopamine D2/D3 receptor radioligand measures is associated with harm avoidant symptoms in anorexia and bulimia nervosa Psych Res Neuroimag 2012 online 10.1016/j.pscychresns.2012.06.010 PMC3880148 23154100

[B24] Bailer UF Bloss CS Frank GK Price JC Meltzer CC Mathis CA Geyer MA Wagner A Becker CR Schork NJ Kaye WH 5-HT1A receptor binding is increased after recovery from bulimia nervosa compared to control women and is associated with behavioral inhibition in both groups Int J Eat Disord 2011 44 477 487 10.1002/eat.20843 20872754 PMC4286242

[B25] Pichika R Buchsbaum MS Bailer UF Hoh C Decastro A Buchsbaum BR Kahy WH Serotonin transporter binding after recovery from bulimia nervosa Int J Eat Disord 2012 45 345 352 10.1002/eat.20944 21671458 PMC3175264

[B26] Bacanu SA Bulik CM Klump KL Fichter MN Halmi KA Keel P Kaplan AS Mitchell JE Rotondo A Strober M Treasure J Woodside DB Sonpar VA Xie W Bergen AW Berretinni WH Kaye WH Devlin B Linkage analysis of anorexia and bulimia nervosa cohorts using selected behavioral phenotypes as quantitative traits or covariates Am J Med Genet 2005 139B 61 68 10.1002/ajmg.b.30226 16152574 PMC2590774

[B27] Bloss C Berrettini W Bergen A Magistretti P Duvvuri V Strober M Brandt H Crawford S Crow S Fichter M Halmi KA Johnson C Kaplan A Keel M Klump K Mitchell J Treasurer J Woodside DB Marzola E Schork M Kaye WH Genetic association of recovery from eating disorders: the role of GABA receptor SNPs Neuropsycholpharm 2011 36 222 2232 10.1038/npp.2011.108 PMC3176559 21750581

[B28] Jacobs MJ Roesch S Wonderlich SA Crosby R Thornton L Wilfley D Berrettini W Brandt H Crowford S Fichter M Halmi KA Johnson C Kaplan AS LaVia M Mitchell JE Rotondo A Strober M Woodside DB Kaye WH Bulik CM Anorexia nervosa trios: behavioral profiles of individuals with anorexia nervosa and their parents Psychol Med 2009 39 451 461 10.1017/S0033291708003826 18578898 PMC3714180

[B29] Wiseman C Sunday SR Klapper F Harris W Halmi KA Changing patterns of hospitalization in eating disorder patients Int J Eat Dis 2001 30 61 74 10.1002/eat.1055 11439410

[B30] Cassano G Miniati M Pini S Rotondo A Banti S Borri C Camilleri V Mauri M 6-Month open trial of haloperidol as an adjunctive treatment for anorexia nervosa: a preliminary report Int J Eat Dis 2003 33 172 177 10.1002/eat.10130 12616583

[B31] Mehler-wex C Ronanos M Kircheiner J Schulze U A typical antipsychotic in severe anorexia nervosa in children and adolescents – review and case reports Eur Eat Dis Rev 2008 16 100 108 10.1002/erv.843 18000964

[B32] Bissada H Tasca G Barber AM Bradwejn J Olanzapine in the treatment of low body weight and obsessive thinking in women with anorexia nervosa: a randomized, double-blind, placebo controlled trial Am J Psychiat 2008 165 1281 1288 10.1176/appi.ajp.2008.07121900 18558642

[B33] Safer D Arnow K Super threshold duloxetine for treatment – resistant depression, anorexia nervosa binge-purging type and obsessive compulsive disorder: a case report Innov Clin Neurosci 2012 9 13 16 22567604 PMC3342990

[B34] Fairburn CG Transdiagnostic cognitive behavior therapy for eating disorders 2010 New York: Guilford Press

[B35] Tchanturia K Morris R Brecelj-Anerluh M Set shifting in anorexia nervosa: an examination of before and after weight gain, in full recovery in relationship to child and adult OCPD J Psychiat Res 2004 38 545 552 10.1016/j.jpsychires.2004.03.001 15380406

[B36] Money C Davies H Tchanturia K A case study introducing cognitive remediation and emotion skills training for anorexia nervosa inpatient care Clin Case Studies 2011 10 110 121 10.1177/1534650110396545

[B37] Wade T Treasurer J Schmidt U Case series evaluation of the maudsley model for treatment of adults with anorexia nervosa Eur Eat Dis Rev 2011 19 382 389 10.1002/erv.1078 21280166

[B38] Williams KD Doney T Geller J Setting the eating disorder aside: an alternative model of care Eur Eat Dis Rev 2010 18 90 96 10.1002/erv.989 20099264

[B39] McIntosh VW Jordan J Luty SE Specialist supportive clinical management for anorexia nervosa Int J Eat Dis 2006 39 625 632 10.1002/eat.20297 16937382

[B40] Touyz S LeGrange D Lacey H A randomized control trial of non specific supportive clinical management versus cognitive behavioral therapy in long standing anorexia nervosa Aus New Zeal J Psychiat 2009 43 suppl A13

[B41] Strober M Managing the chronic, treatment – resistant patient with anorexia nervosa Int J Eat Dis 2004 36 245 255 10.1002/eat.20054 15478130

[B42] Vanderlinden J Many roads lead to Rome Why does cognitive behavioral therapy remain unsuccessful for many eating disorder patients? Eur Eat Dis Rev 2008 16 329 333 10.1002/erv.889 18666311

[B43] Mitchell JE Agras S Crow S Halmi K Fairburn CG Bryson S Kraemer H Step-care and cognitive behavioral therapy for bulimia nervosa: randomized trial Brit J Psychiat 2011 198 391 397 Online March 17 10.1192/bjp.bp.110.082172 21415046 PMC3093678

[B44] Martinez-Mallen E Castro Forniel ESJ Lazaro L Moreno E Morer A Font E Julien J Vila M Torro J Cue exposure in the treatment of resistant adolescent bulimia nervosa Int J Eat Dis 2007 40 596 601 10.1002/eat.20423 17607695

[B45] Carter WP Hudson J LaLonde JK Pindyck L Pharmacological treatment of binge eating disorder Int J Eat Dis 2003 34 74 88 10.1002/eat.10207 12900988

[B46] Kaye W Neurobiology of anorexia and bulimia nervosa Physiol Beh 2008 94 121 135 10.1016/j.physbeh.2007.11.037 PMC2601682 18164737

